# “Your Journey Through Crofton Ward” – A Visual Aid for Patients of Their Pathway Through an Acute Male Unit Within a Medium Secure Setting at a Specialist Forensic Mental Health Service

**DOI:** 10.1192/bjo.2026.11417

**Published:** 2026-06-30

**Authors:** Amy Legister

**Affiliations:** Oxleas NHS Foundation Trust, London, United Kingdom. South London and Maudsley NHS Foundation Trust, London, United Kingdom

## Abstract

**Aims:**

The aim was to create a visual representation of a patient's journey through an acute male unit within a medium secure setting at a specialist forensic mental health service. This poster would also be accompanied by leaflets about specific topics that patients can request if they would like further information. I noticed that many patients admitted onto the ward were asking the same questions to me and other staff during their ward rounds which identified an unmet need. Patients did not have a resource to find out answers or know who best to approach, so I wanted to resolve this. I aimed to improve patient experience on the ward which would improve outcomes and reduce frustration, the risk of getting incorrect information, aggression and complaints.

**Methods:**

I organised focus groups with both patients and staff from each discipline of the Multidisciplinary Team to gather qualitative data about this topic, including thoughts on the idea of the poster itself, and what kind of information they would want included. I frequently joined patient Community Meetings and provided versions of the poster before it was printed so that they could continue to provide their opinions and contributions so it would be co-produced and to ensure the information was relevant.

**Results:**

Once the poster was on display on the ward, I organised further focus groups to collect qualitative data again from both patients and staff. The results were overwhelmingly positive. Some feedback from patients included comments such as “It is easy to understand”, “It is helpful to have a visual poster that I can refer to without having to ask staff”. Feedback from staff included comments such as “Great visuals and really clear to follow”.

**Conclusion:**

This poster has so far had a positive outcome for both patients and staff. For patients it has helped to reduce confusion when admitted onto the ward and frustration regarding the ward processes and their next steps. They have felt more empowered in the knowledge that they can seek answers to commonly asked questions of their own accord. For staff it has allowed ward rounds to be more directed and they feel their role is more understood. The project has scope and is potentially in the process of being replicated in other wards within the service, and could also be replicated in other units nationally so that patients would be able to have a similar experience.

